# Role of P2X7 Receptor on Hypoxia-Induced Vascular Endothelial Growth Factor Gene Expression in H9c2 Rat Cardiomyocytes

**DOI:** 10.3390/jcdd12110438

**Published:** 2025-11-06

**Authors:** Anfal F. Bin Dayel, Reem M. Alhejji, Asma S. Alonazi, Nouf M. Alrasheed

**Affiliations:** Department of Pharmacology and Toxicology, College of Pharmacy, King Saud University, Riyadh 11451, Saudi Arabia; 443203712@student.ksu.edu.sa (R.M.A.); aaloneazi@ksu.edu.sa (A.S.A.); nrasheed@ksu.edu.sa (N.M.A.)

**Keywords:** ischemic heart disease, purinergic P2X7 receptors, vascular endothelial growth factor, hypoxia-inducible factor-1α

## Abstract

Purinergic P2X7 receptors (P2X7Rs) may provide cardioprotection against ischemic heart disease. Cardiac angiogenesis is an endogenous adaptive response of hypoxic cardiomyocytes, mediated by vascular endothelial growth factor (VEGF) via hypoxia-inducible factor-1α (HIF-1α). The study aimed to determine whether P2X7Rs can regulate cardiac pro-angiogenic signaling in hypoxic H9c2 cardiomyocytes by modulating the angiogenic factor VEGF through HIF-1α genes. H9c2 rat cardiomyocytes were exposed to hypoxia alone or in combination with the P2X7R antagonist A740003. Subsequently, ATP levels and LDH activity were measured. The expression of P2X7R, HIF-1α, and VEGF was detected. Intracellular ATP level was significantly lower in hypoxia cardiomyocytes, whereas extracellular ATP, HIF-1α, and LDH levels were significantly higher in hypoxic cardiomyocytes. These effects were associated with increased P2X7R and VEGF gene expressions. Pretreatment with A740003 reversed HIF-1α and VEGF expressions in hypoxic cardiomyocytes. The findings suggest that P2X7Rs regulate pro-angiogenic signaling in hypoxic cardiomyocytes through the HIF-1α/VEGF pathway. Thus, the P2X7R-mediated HIF-1α/VEGF pathway may represent a novel approach to stimulating angiogenesis and preventing heart failure in ischemic heart disease.

## 1. Introduction

Ischemic heart disease is a serious condition marked by insufficient oxygen reaching cardiomyocytes and reduced blood flow to the myocardium [1]. Prolonged oxygen deprivation in this condition can accelerate cardiomyocyte injury, deteriorating cardiac structure and function [2]. However, when cardiomyocytes are hypoxic, several metabolic and hemodynamic compensatory mechanisms may emerge [3,4], potentially promoting cell regeneration and preventing heart failure. Indeed, the molecular mechanisms underlying compensatory mechanisms in hypoxic cardiomyocytes have not yet been fully elucidated, especially cardiac angiogenesis as an endogenous adaptive response to cardiac ischemia.

Cardiac angiogenesis, the process by which new blood vessels develop from pre-existing ones, is an essential compensatory mechanism in hypoxia cardiomyocyte injuries [5]. The transcription factor hypoxia-inducible factor-1α (HIF-1α) plays a role in regulating cardiac angiogenesis. HIF-1α is hydrolyzed by the enzyme prolyl hydroxylase under normal oxygen conditions. This enzyme is inhibited when oxygen levels are low, leading to the accumulation of HIF-1α and stimulation of pro-angiogenic vascular endothelial growth factor (VEGF) [6]. In ischemic heart disease, hypoxic cardiomyocytes stimulate cardiac angiogenesis by activating the HIF-1α/VEGF signaling pathway [7]. The stimulation of cardiac angiogenesis can protect the myocardium from developing heart failure in the early stages after myocardial ischemia by promoting the formation of new capillaries and restoring blood supply to the ischemic area [8]. However, prolonged hypoxia in cardiomyocytes can inhibit angiogenesis by activating p53, a tumor suppressor protein that degrades HIF-1α. Inhibition of cardiac angiogenesis has been linked to deterioration in cardiac function and the development of heart failure [7]. Therefore, identifying therapeutic targets that promote cardiac angiogenesis in early stage of myocardial ischemia is critical for developing novel heart failure prevention strategies.

Hypoxic cardiomyocytes can stimulate the release of adenosine triphosphate (ATP) into the extracellular space. Accumulation of ATP in the extracellular space activates purinergic P2X7 receptors (P2X7Rs), opening ion channels that support Na^+^ and Ca^2+^ influx and K^+^ efflux [9,10,11]. Studies have shown that activating P2X7Rs can reduce ischemia-induced cardiomyocyte damage by increasing the release of cardioprotective substances like adenosine [12,13]. The beneficial effect of P2X7Rs in myocardial ischemia has been confirmed by P2X7R antagonists, such as brilliant blue G, which increase infarction size and inhibit cardioprotective mechanisms [13]. Interestingly, P2X7R expression is upregulated in response to myocardial ischemia [14,15]. Upregulation of P2X7R expression during myocardial ischemia can reduce apoptosis and cardiomyocyte injury by increasing phosphorylation of extracellular signal-regulated kinase 1/2 (ERK1/2) [14,16]. Although research has shown that upregulating P2X7Rs can protect against myocardial ischemia injury, the precise mechanisms underlying their regulation of cardiac angiogenesis during myocardial ischemia are unknown. As a result, we proposed that P2X7Rs regulate pro-angiogenic factor in hypoxic H9c2 cardiomyocytes by upregulating VEGF gene expression in an in vitro hypoxia model. We examined VEGF and HIF-1α gene expression to evaluate the effect of P2X7R antagonist A740003 on the angiogenic signaling pathway induced by hypoxia. This study advances our understanding of cardiac angiogenesis as a compensatory mechanism in response to ischemia, particularly in the early stages of hypoxic cardiomyocytes.

## 2. Materials and Methods

### 2.1. Drugs and Kits

A740003 and Echinomycin were procured from MedChemExpress (Monmouth Junction, NJ, USA). The ENLITEN^®^ ATP Bioluminescent assay kit was obtained from Promega (Madison, WI, USA). The Lactate Dehydrogenase (LDH) kit and Cell Counting Kit-8 (CCK-8) were obtained from MedChemExpress (Monmouth Junction, NJ, USA). The rat-specific enzyme-linked immunosorbent assay (ELISA) kit for VEGF was obtained from Solarbio Life Science (Beijing, China).

### 2.2. Cell Culture

Rat embryonic cardiomyocyte (H9c2) cells obtained from the American Tissue Type Collection (Manassas, VA, USA; CRL-1446) were seeded in Dulbecco’s Modified Eagle’s Medium (DMEM) with 1% penicillin–streptomycin solution and 10% fetal bovine serum (FBS). H9c2 cells were cultured to approximately 80% confluence at 37 °C under normoxic conditions of 21% O_2_ and 5% CO_2_.

### 2.3. Induction of Hypoxia

**Figure 1:**
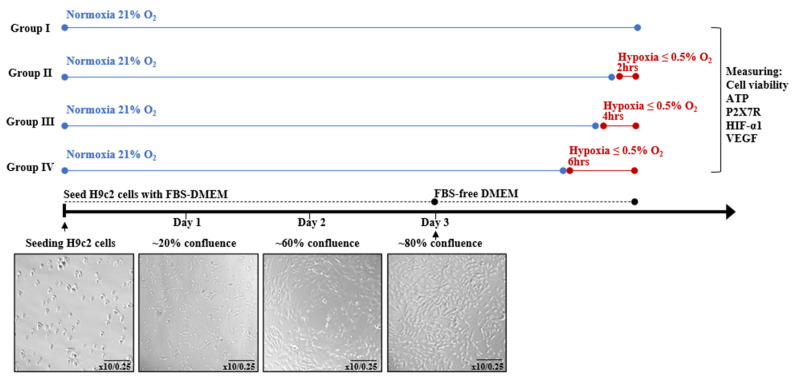
Induction of hypoxia in H9c2 cardiomyocytes for different durations.

H9c2 cells were exposed to hypoxia, when these cells were at approximately 80% confluence, using FBS-free DMEM and vacuum sealing method with ≤0.5% O_2_ and 5% CO_2_ at 37 °C [17]. Initially, H9c2 cells were exposed to different durations of hypoxia (2, 4 and 6 h) to determine the appropriate hypoxic condition for the following experimental design (Figure 1).

### 2.4. Experimental Design

**Figure 2:**
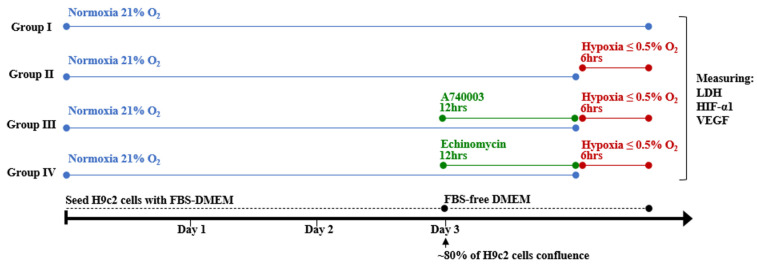
Experimental design for treating H9c2 cardiomyocytes.

H9c2 cells were divided into four groups as follows: Group 1 (normoxia control group), H9c2 cells were incubated under normal conditions. Group 2 (hypoxic control group), H9c2 cells were exposed to hypoxia for the indicated duration. Group 3 (A740003-treated hypoxia group), H9c2 cells were pretreated with 5 μM of A740003 for 12 h [18] and then exposed to hypoxia for the indicated duration [18]. Group 4 (Echinomycin-treated hypoxia group), H9c2 cells were pretreated with 1 nM of Echinomycin for 12 h then exposed to hypoxia for the indicated duration [19]. At the end of treatment, cells were harvested for biochemical and molecular studies (Figure 2). This experimental design was repeated at least three times (*n* ≥ 3).

### 2.5. Determination of Cell Viability

A CCK-8 kit was used to measure cell viability in accordance with the manufacturer’s guidelines. Initially, H9c2 cells were cultured in a 96-well plate at 1 × 10^4^ cells/well containing 100 μL FBS-media and incubated under nomoxia conditions at 21% O_2_, 5% CO_2_ and 37 °C. After 24 h incubation, the FBS-media were changed to FBS-free media and then the cells were exposed to either normoxia or hypoxia conditions for different durations (0, 2, 4 and 6) hours. Then, 10 μL of the CCK-8 buffer was added in each well of the plate and incubated for 1–4 h. A microplate reader was used to measure absorbance at a wavelength 450 nm.

### 2.6. Determination of Cellular ATP

Total extracellular levels of ATP in cell supernatants and intracellular levels of ATP in H9c2 cells were measured with a bioluminescent assay kit using SpectraMax^®^ M5 Multi-Mode Microplate Reader (Molecular Devices, San Jose, CA, USA). H9c2 cells were cultured to approximately 80% confluence in FBS-DMEM into 6 well-plates under normoxia conditions. The FBS-DMEM was then changed to FBS-free DMEM and H9c2 cells were incubated under either normoxia or hypoxia conditions for different durations (0, 2, 4 and 6) hours. To detect extracellular levels of ATP, 1 mL of cell supernatants were transferred into tubes and centrifuged at 12,000 rpm at 4 °C for 10 min [20]. After cell supernatants were transferred, H9c2 cells were treated with 0.5% trichloroacetic acid extraction to detect intracellular levels of ATP. 100 μL of standards and samples were added in a 96 opaque-well plate with 100 μL of Luciferase/Luciferin reagent. The extracellular and intracellular levels of ATP in the samples were then identified by comparing the luminescence of samples to the ATP standard curve.

### 2.7. Determination of Lactate Dehydrogenase Cytotoxicity

LDH activity was measured in cell culture supernatants using a cytotoxicity LDH assay kit. Briefly, H9c2 cells were grown in 96-well plates and treated according to the previous experimental design. To determine the maximum LDH release (used as high control), cell supernatants were mixed with 10 μL lysis buffer. To determine the spontaneous LDH release (used as low control), cell supernatants were mixed with 10 μL FBS-free DMEM. After 30 min incubation at 37 °C, 50 μL of cell supernatants were then transferred into new 96-well plate. The working solution (50 μL) was added and incubated at 25 °C for 30 min in a light-protected environment, followed by the stopping solution (50 μL). A microplate reader was used to measure absorbance value of LDH at a wavelength 490 nm. The percentage of LDH activity in each sample was determined using the following equation: LDH activity (%) = [(absorbance of sample-absorbance of low control)]/[(absorbance of high control-absorbance of low control)] × 100%.

### 2.8. Determination of Pro-Angiogenic Factor VEGF Concentration

VEGF levels in cell culture supernatants were measured using a rat immunoassay ELISA kit. Standard and samples were transferred into a 96-well plate precoated monoclonal antibody specific to VEGF and the immunoassay was performed in accordance with the manufacturer’s guidelines. Briefly, each well was filled with 100 μL of standards or samples and then incubated at 37 °C. After incubating for 90 min, the solution was discarded, and washing buffer was used to rinse the plate. Each well was then incubated with a 100 μL working solution of biotin-conjugated anti-rat VEGF antibody at 37 °C. After 60 min of incubation, the plate was washed before being filled with 100 μL of Streptavidin-HRP working solution. Following 30 min of incubation, the plate was rinsed, then 100 μL of substrate solution was added and incubated at 37 °C for 15 min. The reaction was halted using 50 μL of stop solution. The absorbance was measured at 450 nm with a microplate reader. A recombinant VEGF standard curve was then used to determine the concentration of VEGF in the cell supernatants.

### 2.9. Real-Time Polymerase Chain Reaction (RT-PCR) Analysis

The mRNA expression levels of P2X7R, HIF-1α and VEGF were measured using RT-PCR analysis. Initially, total RNA was extracted from H9c2 cells using PureLink^TM^ RNA Mini Kit (Ambion^®^ by Life Technologies^TM^, Carlsbad, CA, USA) according to the manufacturer’s specifications. The extracted of RNA was then reverse-transcribed into complementary DNA using Reverse Transcription Kit (Applied Biosystems™, Foster, CA, USA) according to the manufacturer’s specifications. Briefly, 1 µg of total RNA was mixed with reverse transcriptase, random primers, RNase inhibitor, dNTPs, and reaction buffer in a total volume of 20 µL. RT-PCR analysis was performed using SYBR^®^ Green Supermix (Bio-Rad, Hercules, CA, USA) on a thermocycler. Forward and reverse primers were obtained from Macrogen with the sequences as follows: P2X7R forward, 5′-CTTCGGCGTGCGTTTTG-3′ and reverse, 5′-AGGACAGGGTGGATC-3′ [21]; HIF-1α forward, 5′-GAAAGGATTACTGAGTTGATGG-3′ and reverse 5′-CAGACATATCCACCTCTTTTTG-3′ [19]; VEGF forward, 5′-ACTTTCTGCTGTCTTGGATG-3′ and reverse 5′-CTCGGCTTGTCACATCACCG-3′ [22]; GAPDH forward, 5′-CTCTGCTCCTCCTGTTCGAC-3′ and reverse 5′-GCGCCCAATACGACCAAATC-3′ [23]. The cycle protocol begins at 50 °C for 2 min, then increases to 95 °C for 10 min, followed by 40 cycles of 95 °C for 15 s and 60 °C for 1 min. GAPDH was used as an internal reference to normalize the mRNA expression level. Relative P2X7R, HIF-1α and VEGF mRNA expressions were expressed as fold change using the 2^−ΔΔthreshold cycle^ method.

### 2.10. Data Analysis

Data analyses were processed using GraphPad Prism version 9.0 (GraphPad Software, Inc., San Diego, CA, USA). Results are presented as mean ± standard error of the mean (SEM). One-way analysis of variance (ANOVA) was used to determine differences between groups, followed by Dunnett’s or Tukey’s multiple comparisons test. Group differences that were statistically significant were indicated by *p*-values ˂ 0.05.

## 3. Results

### 3.1. Hypoxia Induces Cellular Injury in H9c2 Cardiomyocytes

**Figure 3:**
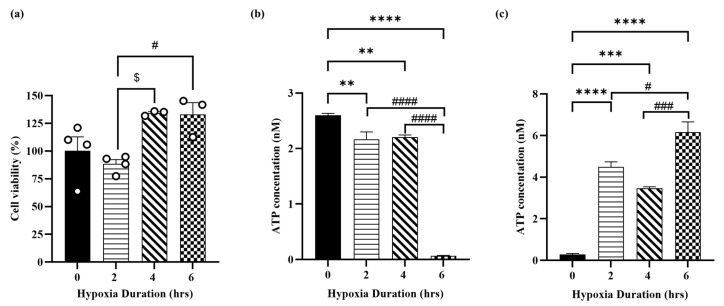
Hypoxia induces cellular injury in H9c2 cardiomyocytes. (**a**) % of H9c2 cell viability under nomoxia condition and different durations of hypoxia (2, 4 and 6 h); (**b**) intracellular and (**c**) extracellular ATP concentrations in H9c2 cardiomyocytes under nomoxia condition and different durations of hypoxia (2, 4 and 6 h). Data are presented as mean ± SEM (*n* ≥ 3). One-way analysis of variance (ANOVA) test was used followed by Tukey’s multiple comparisons test. ** *p* < 0.01, *** *p* < 0.001 and **** *p* < 0.0001 compared to normoxia control group. ^#^ *p* < 0.05, ^###^ *p* < 0.001 and ^####^ *p* < 0.0001 compared to 6 h hypoxia group. ^$^ *p* < 0.05 compared between 2 and 4 h of hypoxia.

Cell viability was determined to show the effect of hypoxia in H9c2 cardiomyocytes. Figure 3a shows that exposing H9c2 cells to 2, 4, and 6 h of hypoxia had no significant effect on cell viability compared to normoxia, with a slight increase in cell viability at 4 and 6 h compared to 2 h of hypoxia. Intracellular and extracellular ATP levels were measured to validate the hypoxia-induced cellular cardiomyocyte injury model. As shown in Figure 3b, exposure of H9c2 cells to hypoxia for 2, 4 and 6 h resulted in decreased intracellular ATP levels with the lowest concentration observed at 6 h. Whereas, exposure of H9c2 cells to hypoxia for 2, 4 and 6 h resulted in a sustained increase in extracellular ATP levels with the highest concentration observed at 6 h (Figure 3c). Figure 3b,c show marked differences in intracellular and extracellular ATP concentrations after 6 h of hypoxia versus 2 and 4 h of hypoxia.

### 3.2. Hypoxia Induces Molecular Injury in H9c2 Cardiomyocytes

**Figure 4:**
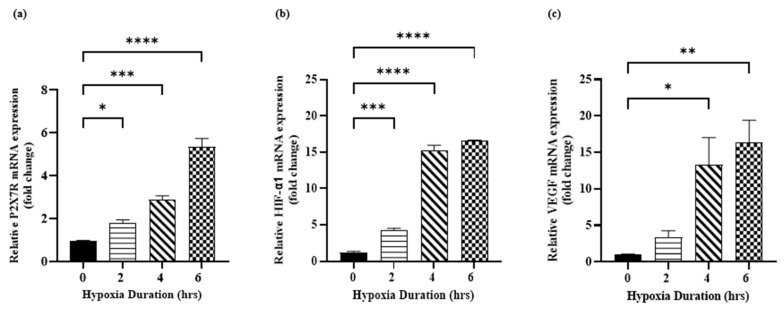
Hypoxia induces molecular injury in H9c2 cardiomyocytes. (**a**) P2X7R; (**b**) HIF-1α and (**c**) VEGF mRNA expression levels in H9c2 cardiomyocytes under normoxia condition and different duration of hypoxia (2, 4 and 6 h). Data are presented as mean ± SEM (*n* ≥ 3). One-way analysis of variance (ANOVA) test was used followed by Dunnett’s multiple comparisons test. * *p* < 0.05, ** *p* < 0.01, *** *p* < 0.001 and **** *p* < 0.0001 compared to normoxia control group.

Exposure of H9c2 cells to hypoxia for 2, 4 and 6 h increased P2X7R mRNA expression by ~2-fold, 3-fold and 5-fold, respectively, compared to the normoxia control group (Figure 4a). The HIF-1α mRNA expression was determined as a marker of hypoxia in H9c2 cardiomyocytes. As shown in Figure 4b, exposure of H9c2 cells to hypoxia for 2, 4 and 6 h significantly increased the HIF-1α mRNA expression by ~4-fold, 15-fold and 16.5-fold, respectively. The VEGF mRNA expression was determined as a marker of pro-angiogenic growth factor in H9c2 cardiomyocytes. Exposure of H9c2 cells to hypoxia for 2, 4 and 6 h significantly increased the VEGF mRNA expression by ~3-fold, 13-fold and 16-fold, respectively, compared to the normoxia control group (Figure 4c). Our results show that the cellular and molecular changes in H9c2 cells were severe when H9c2 cardiomyocytes were exposed to hypoxia for 6 h. Therefore, the duration of hypoxia was set to be 6 h in the subsequent experiments.

### 3.3. Effect of P2X7R Antagonist on Hypoxia-Induced Cytotoxicity in H9c2 Cells

**Figure 5:**
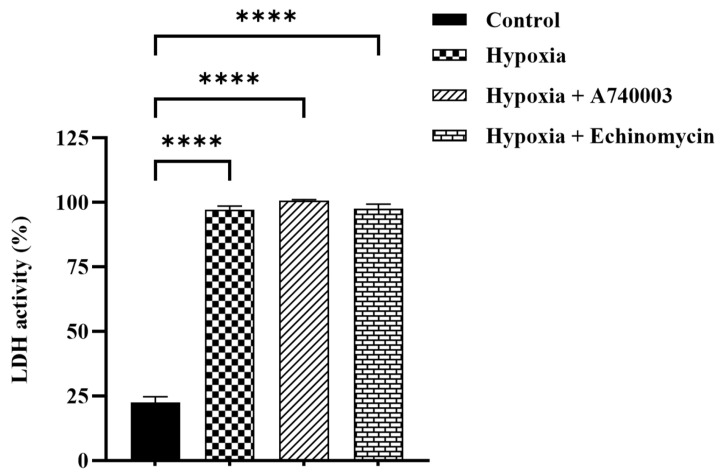
Effect of A740003 and Echinomycin treatment on cytotoxic activity in H9c2 cells exposed to hypoxia. LDH activity in H9c2 cells exposed to 6 h of hypoxia, 5 μM A740003 + hypoxia and 1nM Echinomycin + hypoxia. Data are presented as mean ± SEM (*n* ≥ 3). One-way analysis of variance (ANOVA) test was used followed by Tukey’s multiple comparisons test. **** *p* < 0.0001 compared to normoxia control group.

The LDH was measured as a marker of cytotoxic activity in H9c2 cardiomyocytes. As shown in Figure 5 the cytotoxicity was significantly increased by hypoxic injury in H9c2 cardiomyocytes (*p* < 0.0001). This result was seen when hypoxic cardiomyocytes were pretreated with A740003 and Echinomycin (*p* < 0.0001).

### 3.4. P2X7R Antagonist Inhibits Hypoxia-Induced Pro-Angiogenic Factor Signaling in H9c2 Cells

**Figure 6:**
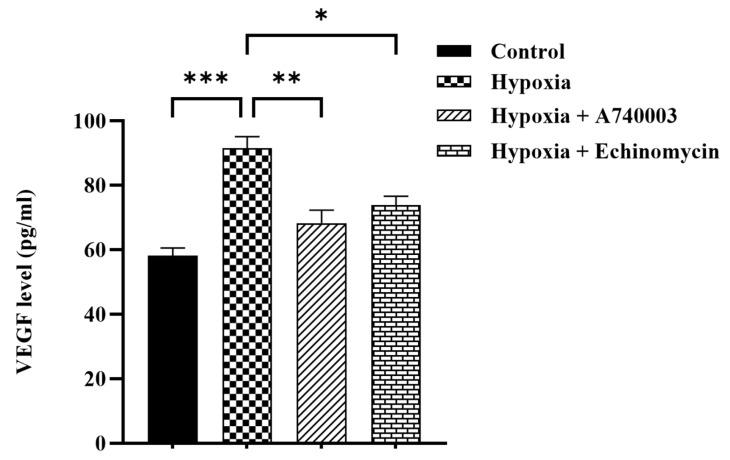
Effect of A740003 and Echinomycin treatment on VEGF level in H9c2 cells exposed to hypoxia. VEGF levels in H9c2 cardiomyocytes exposed to 6 h of hypoxia, 5 μM A740003 + hypoxia and 1nM Echinomycin + hypoxia. Data are presented as mean ± SEM (*n* ≥ 3). One-way analysis of variance (ANOVA) test was used followed by Tukey’s multiple comparisons test. *** *p* < 0.001 compared to normoxia control group; * *p* < 0.05 and ** *p* < 0.01 compared to hypoxia group.

**Figure 7:**
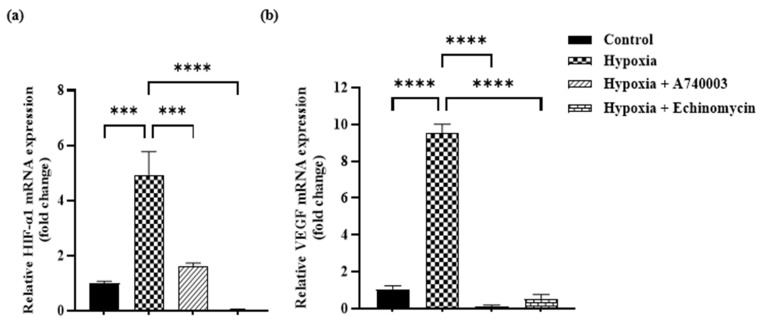
Effect of A740003 and Echinomycin treatment on HIF-1α and VEGF mRNA expression level in H9c2 cells exposed to hypoxia. (**a**) HIF-1α and (**b**) VEGF mRNA expression levels in H9c2 cardiomyocytes exposed to 6 h of hypoxia, 5 μM A740003 + hypoxia and 1nM Echinomycin + hypoxia. Data are presented as mean ± SEM (*n* ≥ 3). One-way analysis of variance (ANOVA) test was used followed by Tukey’s multiple comparisons test. *** *p* < 0.001 and **** *p* < 0.0001 compared to normoxia control group; *** *p* < 0.001 and **** *p* < 0.0001 compared to hypoxia group.

We studied whether A740003 might reduce pro-angiogenic factors in hypoxia cardiomyocytes and investigated potential underling mechanisms. Therefore, HIF-1α and VEGF expression levels were examined. According to ELISA results, VEGF level was significantly increased by ~36.5% in the hypoxia cardiomyocytes compared to that in the normoxia cardiomyocytes (*p* < 0.001). Treatment of hypoxic cardiomyocytes with A740003 and Echinomycin significantly reduced VEGF expression by ~34% and 24%, respectively, as compared to hypoxia group (Figure 6). To determine the effect of A740003 on HIF-1α and VEGF mRNA expression levels in hypoxic cardiomyocytes, we performed RT-PCR analysis. Figure 7a,b show that hypoxia significantly increased HIF-1α and VEGF mRNA expression by ~5-fold and 9.5-fold compared to the normoxia control group (*p* < 0.001 and *p* < 0.0001, respectively). A740003 treatment reduced the increase in HIF-1α and VEGF mRNA expression level in hypoxic cardiomyocytes (*p* < 0.001 and *p* < 0.0001, respectively). Likewise, Echinomycin treatment inhibited the increase in HIF-1α and VEGF mRNA expression level in hypoxic cardiomyocytes compared to the hypoxia group (*p* < 0.0001).

## 4. Discussion

Inadequate oxygen delivery to cardiomyocytes and decreased blood flow to the myocardium are the hallmarks of cardiac ischemia [1]. In this study, we first created a model of cardiac ischemia by exposing H9c2 cells to hypoxia and serum starvation. This in vitro model is known to resemble cardiac ischemia in vivo, causing cellular and molecular changes in cardiomyocytes [24]. Successful hypoxia induction was confirmed by measuring ATP levels, HIF-1α gene expression, and LDH activity in H9c2 cells. ATP is significantly released during cardiac ischemia [25]. Our findings showed that H9c2 cardiomyocytes exposed to hypoxia and serum starvation had significantly higher extracellular ATP levels, with the highest effect observed after 6 h of hypoxia. High extracellular ATP levels were associated with lower intracellular ATP levels, which was consistent with previous studies [19,20,25]. This response was time-dependent, with significant changes in intracellular and extracellular ATP concentrations observed at 6 h of hypoxia compared to 2 and 4 h of hypoxia, possibly due to cumulative metabolic responses within 6 h of sustained oxygen deprivation. The observed inverse relationship between intracellular and extracellular ATP levels in hypoxic H9c2 cells is most likely due to enhanced ATP release from intracellular vesicles across the plasma membrane to the extracellular region [20]. HIF-1α levels reportedly accumulate during cardiac ischemia [26]. Our findings confirmed that H9c2 cardiomyocytes exposed to hypoxia and serum starvation increased HIF-1α gene expression, which is consistent with previous studies [17,19]. Hypoxic H9c2 cells may increase HIF-1α gene expression by inhibiting the prolyl hydroxylase enzyme that degrades HIF-1α [6]. The hypoxia model was confirmed by increased LDH activity in H9c2 cells, which is consistent with previous studies [27]. These findings point to the successful induction of hypoxia in H9c2 cells.

HIF-1α in cardiomyocytes may directly prevent ischemia-induced cell death [28]. Consistent with this, the current study found that overexpressing HIF-1α in cardiomyocytes can prevent hypoxia-induced cell death and maintain cell viability for up to 6 h of hypoxia. Upregulation of HIF-1α during the early stages of hypoxia promotes cell viability by activating VEGF, a growth factor involved in angiogenesis. This is consistent with a previous study showing that upregulation of HIF-1α in acute myocardial infarction can prevent cardiomyocyte apoptosis by increasing the antioxidant response [29]. Despite its protective role against ischemic injury, prolonged high levels of HIF-1α have been shown to cause cardiac rupture through tumor suppressor protein p53, an HIF-1α degradation factor-dependent apoptosis following myocardial infarction [30]. Furthermore, previous research indicates that prolonged hypoxia with high levels of HIF-1α induces cardiomyocyte death through activating pro-apoptotic genes such as BNIP3 [31]. HIF-1α appears to play both beneficial and harmful roles depending on its expression level. High levels of HIF-1α can prevent cardiomyocyte death during early hypoxia, but prolonged high levels can cause cardiomyocyte death.

Cardiac angiogenesis is a distinct feature of hypoxic cardiomyocytes, which can protect the myocardium from heart failure in the early stages of ischemia by stimulating the formation of new capillaries and restoring blood supply to the ischemic area [5,8]. Previous research has shown that cardiac ischemia can promote angiogenesis by activating the HIF-1α/VEGF pathway [7,19,32]. Similarly, in the current study, H9c2 cardiomyocytes exposed to hypoxia and serum starvation showed significantly higher levels of the angiogenic factor VEGF, accompanied by upregulation of HIF-1α gene expression. These findings indicate that hypoxic H9c2 cardiomyocytes successfully activate pro-angiogenic signaling through upregulation of the HIF-1α/VEGF pathway.

P2X7R is associated with ischemic heart disease, with elevated levels detected in experimental models of cardiac ischemia. [14,15]. Similarly, in the current study, H9c2 cardiomyocytes exposed to hypoxia and serum starvation showed a significant increase in P2X7R expression. The increase in P2X7R expression could be attributed to ATP accumulation in the extracellular space, which activates the P2X7R. Our data show that short-term hypoxia had no significant effect on the viability of H9c2 cells, indicating a low level of cell injury, which was associated with upregulation of P2X7Rs. Therefore, future studies are required to gain a better understanding of the role of P2X7Rs in hypoxic cardiomyocytes using assays that directly detect cell injury, such as apoptosis or necrosis, mitochondrial function, and ROS production. The current study also observed that upregulation of P2X7R in hypoxic cardiomyocytes was associated with decreased intracellular ATP levels and increased extracellular ATP levels. These changes in cellular ATP levels could be attributed to metabolic responses to oxygen deprivation. Previous studies have suggested mechanistic insights into the potential roles of P2X7R in regulating cellular energy metabolism. For example, Adinolfi et al. (2005) found that prolonged P2X7R activation causes mitochondrial dysfunction, implying that P2X7R can influence ATP production through effects on mitochondria [33]. Furthermore, Di Virgilio et al. (2017, 2018) suggested that P2X7R activation enhances ATP release through the P2X7R pores or pannexin-1 channels, leading to extracellular ATP accumulation [34,35]. These studies are consistent with our study showing that upregulation of P2X7R contributes to the cellular ATP imbalance under hypoxic conditions.

**Figure 8:**
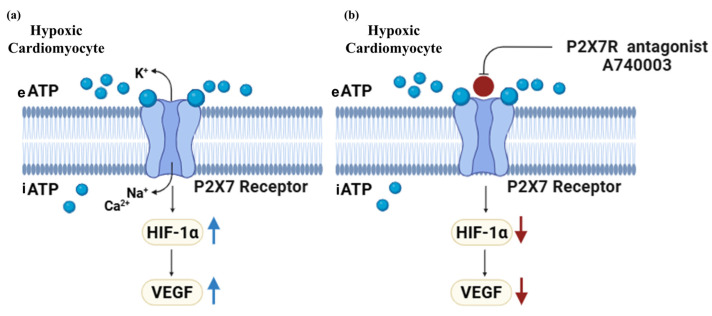
Schematic representation of the role of P2X7Rs in pro-angiogenic signaling in response to hypoxic cardiomyocytes. (**a**) Hypoxic cardiomyocytes activate P2X7R, which stimulates cardiac pro-angiogenic signaling through HIF-1α/VEGF. (**b**) A740003, a P2X7R antagonist, inhibits pro-angiogenic signaling in hypoxic cardiomyocytes by inhibiting the HIF-1α/VEGF pathway. Abbreviations: eATP, extracellular adenosine 5-triphosphate; iATP, intracellular adenosine 5-triphosphate; HIF-1α, Hypoxia-inducible factor 1 alpha; VEGF, vascular endothelial growth factor. Blue circles represent ATP, while the red circle represents the P2X7R antagonist A740003. (Created with BioRender.com).

Previous research demonstrated that P2X7Rs have cardioprotective effects on myocardial ischemia [12,13]. However, the precise mechanism by which P2X7Rs regulate cardiac angiogenesis during cardiac ischemia is unknown. Therefore, we aimed to investigate whether P2X7Rs regulate pro-angiogenic factor signaling in hypoxic cardiomyocytes and elucidate the underlying mechanisms. Our findings were align with a previous study in H9c2 cardiomyocytes [19], which found HIF-1α overexpression in hypoxic H9c2 cardiomyocytes correlates with increased VEGF expression. The current study showed that the increase in HIF-1α and VEGF in hypoxic cardiomyocytes was associated with increased P2X7R expression, suggesting that P2X7R can induce pro-angiogenic signaling in the early stage of cardiac ischemia by activating the HIF-1α/VEGF pathway (Figure 8a). This study extends previous findings indicating that upregulation of P2X7R during myocardial infarction activates the Nox4/PERK/ATF4 pathway [36], which is known to regulate HIF-1α and VEGF [37]. Although upregulation of P2X7Rs has been shown to regulate pro-angiogenic factor VEGF through HIF-1α in early hypoxia, prolonged hypoxia in cardiomyocytes may reverse the role of P2X7R in pro-angiogenic signaling. This is supported by a previous study that found that sustained hypoxia in cardiomyocytes activates the tumor suppressor p53, which impairs angiogenesis by inhibiting HIF-1α/VEGF [7].

Role of P2X7Rs in the pro-angiogenic signaling pathway in early hypoxic cardiomyocytes was confirmed using a P2X7R antagonist. This study used Echinomycin, an HIF-1α inhibitor, to determine if changes in the pro-angiogenic factor signaling during hypoxia were specifically caused by the P2X7R antagonist. Pretreatment of hypoxic H9c2 cardiomyocytes with P2X7R antagonist A740003 inhibited the pro-angiogenic factor VEGF gene and significantly reduced the HIF-1α gene. Consistent with other pathological conditions, Yang et al. found that A740003 inhibits retinal angiogenesis by suppressing HIF-1α/VEGF expression [38], and Zhang et al. found that A740003 inhibits angiogenesis in a cancer model [18]. These results indicate that the P2X7R antagonist modulates the angiogenic signaling in hypoxic cardiomyocytes by inhibiting the P2X7R-mediated HIF-1α/VEGF pathway (Figure 8b). Collectively, our data illustrate that P2X7Rs promote pro-angiogenic signaling in early hypoxic cardiomyocytes by regulating the HIF-1α/VEGF pathway. Interestingly, our results indicated that pretreatment of hypoxic H9c2 cardiomyocytes with P2X7R antagonist A740003 and Echinomycin alone does not effectively reduce LDH activity. This may be attributed to other alternative pathways that kept LDH active under hypoxic conditions.

The current study utilized the rat embryonic H9c2 cell model, a model previously used in cardiac research to study pro-angiogenic signaling involving VEGF and HIF-1α under hypoxic conditions [19,39]. One limitation of the H9c2 cell model is its inability to fully mimic the function of mature cardiomyocytes or endothelial-specific behaviors, such as angiogenesis. Therefore, further validation using endothelial cell models, such as primary cardiac endothelial cells is required. This study demonstrates that P2X7Rs regulate pro-angiogenic signaling in hypoxic cardiomyocytes through the HIF-1α/VEGF pathway, providing indirect evidence for angiogenesis. To confirm the role of P2X7Rs in angiogenesis during hypoxic cardiomyocytes, additional functional angiogenesis assays, such as the tube formation assay, are required, as well as the use of in vivo angiogenesis models, such as the myocardial infarction animal model with left anterior descending (LAD) artery ligation. Another limitation of this study is the lack of analysis of protein expression levels, indicating the need for further validation using Western blotting to confirm P2X7R-mediated through the HIF-1α/VEGF pathway in hypoxic cardiomyocytes. Interestingly, our findings revealed that cell survival pathways can be activated under hypoxic conditions, implying that more research is needed to assess autophagy-related markers during hypoxic stress in order to better understand cell survival pathways and their interactions with P2X7R signaling.

## 5. Conclusions

P2X7Rs can modulate cardiomyocyte responses in hypoxic environments. The study demonstrates that P2X7Rs regulate pro-angiogenic signaling in hypoxic cardiomyocytes via the HIF-1α/VEGF pathway. This advances our understanding of how angiogenesis can be stimulated, particularly in the early stages of hypoxic cardiomyocytes. Future research that directly detects hypoxia-induced cell injury, including apoptosis or necrosis, mitochondrial function, or ROS production, is required to demonstrate whether the P2X7R-mediated pro-angiogenic signaling response has a cardioprotective effect. Further research is needed to confirm the role of P2X7Rs in hypoxic cardiomyocytes via HIF-1α/VEGF in experimental animal models of cardiac ischemia, such as the LAD artery ligation model.

## Data Availability

The study data are available upon request from the corresponding author.

## Abbreviations

The following abbreviations are used in this manuscript:

ATP	Adenosine triphosphate
CCK-8	Cell Counting Kit-8
DMEM	Dulbecco’s Modified Eagle’s Medium
FBS	Fetal bovine serum
HIF-1α	Hypoxia-inducible factor-1α
LAD	left anterior descending
LDH	Lactate Dehydrogenase
P2X7Rs	Purinergic P2X7 receptors
RT-PCR	Real-Time Polymerase Chain Reaction
SEM	Standard error of the mean
VEGF	Vascular endothelial growth factor

## References

[1] Jensen R.V., Hjortbak M.V., Bøtker H.E. (2020). Ischemic heart disease: An update. Semin. Nucl. Med..

[2] Heusch G. (2020). Myocardial ischaemia–reperfusion injury and cardioprotection in perspective. Nat. Rev. Cardiol..

[3] Yoshida S., Adachi H., Murata M., Tomono J., Oshima S., Kurabayashi M. (2017). Importance of compensatory heart rate increase during myocardial ischemia to preserve appropriate oxygen kinetics. J. Cardiol..

[4] Lekven J., Mjøs O.D., Kjekshus J.K. (1973). Compensatory mechanisms during graded myocardial ischemia. Am. J. Cardiol..

[5] Zimna A., Kurpisz M. (2015). Hypoxia-inducible factor-1 in physiological and pathophysiological angiogenesis: Applications and therapies. Biomed. Res. Int..

[6] Wu X., Reboll M.R., Korf-Klingebiel M., Wollert K.C. (2021). Angiogenesis after acute myocardial infarction. Cardiovasc. Res..

[7] Katagiri M., Yamada S., Katoh M., Ko T., Ito M., Komuro I. (2022). Heart failure pathogenesis elucidation and new treatment method development. JMA J..

[8] Cochain C., Channon K.M., Silvestre J.-S. (2013). Angiogenesis in the infarcted myocardium. Antioxid. Redox Signal..

[9] Bodin P., Burnstock G. (2001). Purinergic signalling: ATP release. Neurochem. Res..

[10] Jiang L.-H., Roger S., Baldwin S. (2013). Insights into the molecular mechanisms underlying mammalian P2X7 receptor functions and contributions in diseases, revealed by structural modeling and single nucleotide polymorphisms. Front. Pharmacol..

[11] Dosch M., Gerber J., Jebbawi F., Beldi G. (2018). Mechanisms of ATP release by inflammatory cells. Int. J. Mol. Sci..

[12] Vessey D.A., Li L., Kelley M. (2010). Pannexin-I/P2X 7 purinergic receptor channels mediate the release of cardioprotectants induced by ischemic pre-and postconditioning. J. Cardiovasc. Pharmacol. Ther..

[13] Vessey D.A., Li L., Kelley M. (2011). Ischemic preconditioning requires opening of pannexin-1/P2X7 channels not only during preconditioning but again after index ischemia at full reperfusion. Mol. Cell. Biochem..

[14] Tu G., Zou L., Liu S., Wu B., Lv Q., Wang S., Xue Y., Zhang C., Yi Z., Zhang X. (2016). Long noncoding NONRATT021972 siRNA normalized abnormal sympathetic activity mediated by the upregulation of P2X7 receptor in superior cervical ganglia after myocardial ischemia. Purinergic Signal..

[15] Gu M., Zheng A.-b., Jin J., Cui Y., Zhang N., Che Z.-p., Wang Y., Zhan J., Tu W.-j. (2016). Cardioprotective effects of genistin in rat myocardial ischemia-reperfusion injury studies by regulation of P2X7/NF-κB pathway. Evid.-Based Complement. Altern. Med..

[16] Wang Y. (2007). Mitogen-activated protein kinases in heart development and diseases. Circulation.

[17] Matthiesen S., Jahnke R., Knittler M.R. (2021). A straightforward hypoxic cell culture method suitable for standard incubators. Methods Protoc..

[18] Zhang Y., Cheng H., Li W., Wu H., Yang Y. (2019). Highly-expressed P2X7 receptor promotes growth and metastasis of human HOS/MNNG osteosarcoma cells via PI3K/Akt/GSK3β/β-catenin and mTOR/HIF1α/VEGF signaling. Int. J. Cancer.

[19] Osuru H.P., Lavallee M., Thiele R.H. (2022). Molecular and Cellular Response of the myocardium (H9C2 cells) towards hypoxia and HIF-1α inhibition. Front. Cardiovasc. Med..

[20] Gerasimovskaya E.V., Ahmad S., White C.W., Jones P.L., Carpenter T.C., Stenmark K.R. (2002). Extracellular ATP is an autocrine/paracrine regulator of hypoxia-induced adventitial fibroblast growth: Signaling through extracellular signal-regulated kinase-1/2 and the Egr-1 transcription factor. J. Biol. Chem..

[21] Ding L., Gong C., Zhao J., Liu X., Li T., Rao S., Wang S., Liu Y., Peng S., Xiao W. (2019). Noncoding transcribed ultraconserved region (T-UCR) UC.48+ is a novel regulator of high-fat diet induced myocardial ischemia/reperfusion injury. J. Cell. Physiol..

[22] Wang S.-y., Zou C., Liu X.-f., Yan Y.-j., Gu S.-z., Li X. (2021). Vascular endothelial growth factor ameliorated palmitate-induced cardiomyocyte injury via JNK pathway. Vitr. Cell Dev. Biol. Anim..

[23] Jiang H., Wang H., Liu T., Yang Z., Zhang R., Han H. (2018). Co-cultured the MSCs and cardiomyocytes can promote the growth of cardiomyocytes. Cytotechnology.

[24] Bonavita F., Stefanelli C., Giordano E., Columbaro M., Facchini A., Bonafè F., Caldarera C.M., Guarnieri C. (2003). H9c2 cardiac myoblasts undergo apoptosis in a model of ischemia consisting of serum deprivation and hypoxia: Inhibition by PMA. FEBS Lett..

[25] Vial C., Owen P., Opie L., Posel D. (1987). Significance of release of adenosine triphosphate and adenosine induced by hypoxia or adrenaline in perfused rat heart. J. Mol. Cell. Cardiol..

[26] Zieseniss A., Hesse A.R., Jatho A., Krull S., Hölscher M., Vogel S., Katschinski D.M. (2015). Cardiomyocyte-specific transgenic expression of prolyl-4-hydroxylase domain 3 impairs the myocardial response to ischemia. Cell. Physiol. Biochem..

[27] Jing L., Li Q., He L., Sun W., Jia Z., Ma H. (2017). Protective effect of tempol against hypoxia-induced oxidative stress and apoptosis in H9c2 cells. Med. Sci. Monit. Basic. Res..

[28] Sato T., Takeda N. (2023). The roles of HIF-1α signaling in cardiovascular diseases. J. Cardiol..

[29] Datta Chaudhuri R., Banik A., Mandal B., Sarkar S. (2021). Cardiac-specific overexpression of HIF-1α during acute myocardial infarction ameliorates cardiomyocyte apoptosis via differential regulation of hypoxia-inducible pro-apoptotic and anti-oxidative genes. Biochem. Biophys. Res. Commun..

[30] Ikeda M., Ide T., Tadokoro T., Miyamoto H.D., Ikeda S., Okabe K., Ishikita A., Sato M., Abe K., Furusawa S. (2021). Excessive hypoxia-inducible factor-1α expression induces cardiac rupture via p53-dependent apoptosis after myocardial infarction. JAHA.

[31] Feng C.-C., Lin C.-C., Lai Y.-P., Chen T.-S., Marthandam Asokan S., Lin J.-Y., Lin K.-H., Viswanadha V.P., Kuo W.-W., Huang C.-Y. (2016). Hypoxia suppresses myocardial survival pathway through HIF-1α-IGFBP-3-dependent signaling and enhances cardiomyocyte autophagic and apoptotic effects mainly via FoxO3a-induced BNIP3 expression. Growth Factors.

[32] Lee S.H., Wolf P.L., Escudero R., Deutsch R., Jamieson S.W., Thistlethwaite P.A. (2000). Early expression of angiogenesis factors in acute myocardial ischemia and infarction. N. Engl. J. Med..

[33] Adinolfi E., Callegari M.G., Ferrari D., Bolognesi C., Minelli M., Wieckowski M.R., Pinton P., Rizzuto R., Di Virgilio F. (2005). Basal activation of the P2X7 ATP receptor elevates mitochondrial calcium and potential, increases cellular ATP levels, and promotes serum-independent growth. Mol. Biol. Cell.

[34] Di Virgilio F., Dal Ben D., Sarti A.C., Giuliani A.L., Falzoni S. (2017). The P2X7 receptor in infection and inflammation. Immunity.

[35] Di Virgilio F., Schmalzing G., Markwardt F. (2018). The elusive P2X7 macropore. Trends Cell Biol..

[36] Zhang S., Bi Y., Xiang K., Tang Y. (2025). P2X7 Receptor Facilitates Cardiomyocyte Autophagy After Myocardial Infarction via Nox4/PERK/ATF4 Signaling Pathway. Cell Biochem. Funct..

[37] Zhang M., Brewer A.C., Schröder K., Santos C.X., Grieve D.J., Wang M., Anilkumar N., Yu B., Dong X., Walker S.J. (2010). NADPH oxidase-4 mediates protection against chronic load-induced stress in mouse hearts by enhancing angiogenesis. Proc. Natl. Acad. Sci. USA.

[38] Yang M., Qiu R., Wang W., Liu J., Jin X., Li Y., Li L., Lei B. (2021). P2X7 Receptor Antagonist Attenuates Retinal Inflammation and Neovascularization Induced by Oxidized Low-Density Lipoprotein. Oxid. Med. Cell. Longev..

[39] Li B., Yu J., Liu P., Zeng T., Zeng X. (2021). Astragaloside IV protects cardiomyocytes against hypoxia injury via HIF-1α and the JAK2/STAT3 pathway. Ann. Transl. Med..

